# Locking compression plate fixation of critical-sized bone defects in sheep. Development of a model for veterinary bone tissue engineering

**DOI:** 10.1590/ACB360601

**Published:** 2021-06-25

**Authors:** Geissiane de Moraes Marcondes, Nicole Fidalgo Paretsis, Anderson Fernando de Souza, Maria Raquel Bellomo Agrello Ruivo, Mário Antônio Ferraro Rego, Fernanda Silveira Nóbrega, Silvia Renata Gaido Cortopassi, André Luis do Valle De Zoppa

**Affiliations:** 1 PhD. Department of Surgery - School of Veterinary Medicine and Animal Science – Universidade de São Paulo - Sao Paulo (SP), Brazil.; 2 PhD. Department of Surgery - School of Veterinary Medicine and Animal Science – Universidade de São Paulo - Sao Paulo (SP), Brazil.; 3 MSc. Department of Surgery - School of Veterinary Medicine and Animal Science – Universidade de São Paulo - Sao Paulo (SP), Brazil.; 4 PhD. Department of Surgery - School of Veterinary Medicine and Animal Science – Universidade de São Paulo - Sao Paulo (SP), Brazil.; 5 PhD. Associate Professor. Department of Surgery - School of Veterinary Medicine and Animal Science – Universidade de São Paulo - Sao Paulo (SP), Brazil; 6 PhD. Associate Professor. Department of Surgery - School of Veterinary Medicine and Animal Science – Universidade de São Paulo - Sao Paulo (SP), Brazil.

**Keywords:** Veterinary Surgery, Animal Models, Orthopedics, Bone Regeneration

## Abstract

**Purpose:**

To develop a segmental tibial bone defect model for tissue engineering studies in veterinary orthopedics using single locking compression plate (LCP) fixation and cast immobilization.

**Methods:**

A 3-cm bone defect was created in the right tibia of 18 adult Suffolk sheep. A 10-hole, 4.5-mm LCP was applied to the dorsomedial aspect of the bone. Four locking screws were inserted into the proximal and three into the distal bone fragment. Operated limbs were immobilized with casts. Animals were submitted to stall rest, but were allowed to bear weight on the operated limb. During the recovery period, animals were checked daily for physiological parameters, behavior and lameness. Follow-up radiographs were taken monthly.

**Results:**

Surgical procedures and postoperative recovery were uneventful. Animals adapted quickly to casts and were able to bear weight on the operated limb with no signs of discomfort or distress. No clinical or radiographic complications were detected over a 90-day follow-up period.

**Conclusions:**

Surgical creation of tibial segmental bone defects followed by fixation with single LCP and cast immobilization was deemed a feasible and appropriate model for veterinary orthopedic research in tissue engineering.

## Introduction

The ovine critical-size defect model is a well-established animal model for bone healing investigation^1^–^3^. Adult sheep are deemed well suited for translational research in biomaterials and bone engineering studies due to their docile temperament, easy handling and structural similarities between ovine and human bone^4^–^7^. Sheep are also used as experimental models in bone tissue engineering studies involving large animals^8^,^9^. Critical-sized bone defects are defects in a particular bone that will not heal spontaneously during the organism lifetime^3^. Critical size of tibial bone defects in ovine models range from 3 to 5 cm^1^–^3^. Full characterization of any given model should provide information regarding defect size and location, bone geometry, whether or not periosteal stripping was performed and weight-bearing loads, as well as detailed description of preoperative and follow-up procedures. Selected fixation method, postoperative management and potential complications should also be detailed^3^,^10^.

Several fracture stabilization methods have been investigated in ovine models, the most common being external skeletal fixation (ESF), internal fixation with intramedullary pins or plate and screws, combination fixation with intramedullary pins and plates and double plating^1^–^3^,^11^. External fixators are user-friendly devices. However, fractures treated with this method take longer to heal, carry a higher risk of infection and are subject to relative instability^1^.

Dynamic compression plates (DCP) are commonly used in ovine tibial segmental bone defect models. In spite of appropriate bending stiffness demonstrated in *ex vivo* biomechanical testings^7^,^12^, DCPs may compromise periosteal blood supply and high screw loosening rates have been reported^1^.

Locking compression plates (LCP) are modern devices which allow biomechanical forces to be transmitted through screw heads. These devices act in a similar fashion to an external fixator, with less periosteal disruption and bone damage^13^. Locking compression plates are widely used in large animal orthopedics, especially in the equine species, given their higher yield strength, greater stiffness and ability to prevent movement at the fracture site^13^,^14^. These plates may also be implanted using minimally invasive techniques, particularly in the distal tibial and femoral fractures^15^. Locking compression plates are equipped with versatile combi-holes specially designed for insertion of locking or cortical screws, at surgeon’s discretion or according to bone region^13^,^16^. They also provide stable fixation and do not interfere with the application of biomaterials, which may be used to enhance bone regeneration^1^.

These plates have been used in combination with different postoperative management strategies (e.g., cast immobilization and suspension devices) for bone defect stabilization in sheep with different rates of complications such as plate bending and fracture^1^,^11^,^17^. However, different from human patients, large animals bear weight on the operated limb immediately after surgery and cast immobilization is often preferred to sling suspension^18^. Bone repair research has attracted increasing attention in the recent past. Ovine experimental bone defect models involving LCP fixation and immediate postoperative weight bearing have seldom been described in literature. Therefore, research in this field of veterinary orthopedics and in bone regeneration in particular is warranted.

This study set out to develop a segmental bone defect model for bone tissue engineering studies in veterinary orthopedics using female sheep. Experimental 3-cm long tibial bone defects were created and stabilized using single LCP fixation and postoperative limb casts. Animals were submitted to clinical and radiographic assessment over a 90-day follow-up period.

## Methods

This study was reviewed and approved by the Ethics Committee on Animal Use of the School of Veterinary Medicine and Animal Science of the Universidade de São Paulo (CEUA/FMVZ - 7100150715), in compliance with the Brazilian National Council for Control of Animal Experimentation (CONCEA) guidelines.

Eighteen (n = 18) empty and orthopedically sound female Suffolk sheep (*Ovis aries*) weighing 50 to 60 kg and aged 3 to 4 years were used. Orthopedic soundness was clinically and radiographically determined. Animals were submitted to a 60-day adaptation period prior to surgical procedures. During the experimental period, animals were housed in pairs in dedicated small ruminant stalls with high sawdust bedding and allowed free access to water, salt (Guabiphós, Pará de Minas, MG, Brazil) and a maintenance diet consisting of Tifton grass and commercial pelleted feed (Ovicorte, Pará de Minas, MG, Brazil). Animals were identified and vaccinated against clostridiosis (3 mL·head^–1^·SC^–1^) (Sintoxan Polivalente, Boehringer Ingelheim Saúde Animal, Paulínia, SP, Brazil) and dewormed with 1% ivermectin (200 µg·kg^–1^·SC^–1^) (Ivomec, Boehringer Ingelheim Saúde Animal, Paulínia, SP, Brazil). Other procedures were as follows: complete blood count, biochemical analysis of liver and kidney function, determination of serum calcium and phosphate levels and FAMACHA scoring.

Surgical procedures were carried out under general anesthesia. Animals were fasted of solid food and water (36 and 24 h, respectively) and submitted to physical examination. A venous catheter was then inserted for premedication with 0.03 mg·kg^–1^ of 2% xylazine hydrochloride (Rompun, Bayer Saúde Animal, São Paulo, SP, Brazil). This was followed by induction with 2 mg·kg^–1^ of propofol (Propovan, Cristália, Itapira, SP, Brazil) combined with 1 mg·kg^–1^ of ketamine hydrochloride (Cetamin, Syntec, Santana de Parnaíba, SP, Brazil) and maintenance with 100% isoflurane (Isoforine, Itapira, SP, Brazil) vaporized in oxygen. Animals showing 20% increase in blood pressure, heart rate and respiratory rates relative to baseline over the course of surgery received 2.5 µg·kg^–1^ of fentanyl citrate (Fentanest, Cristália, Itapira, SP, Brazil).

**Figure 1:**
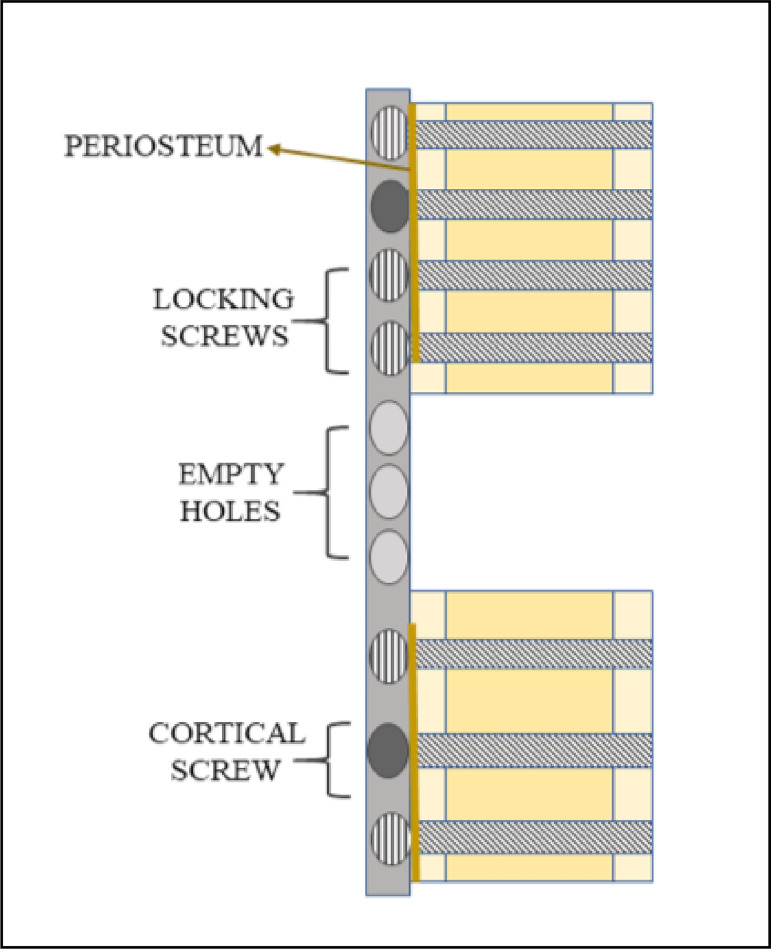
Locking compression plate fixation of a segmental tibial bone defect.

**Figure 2:**
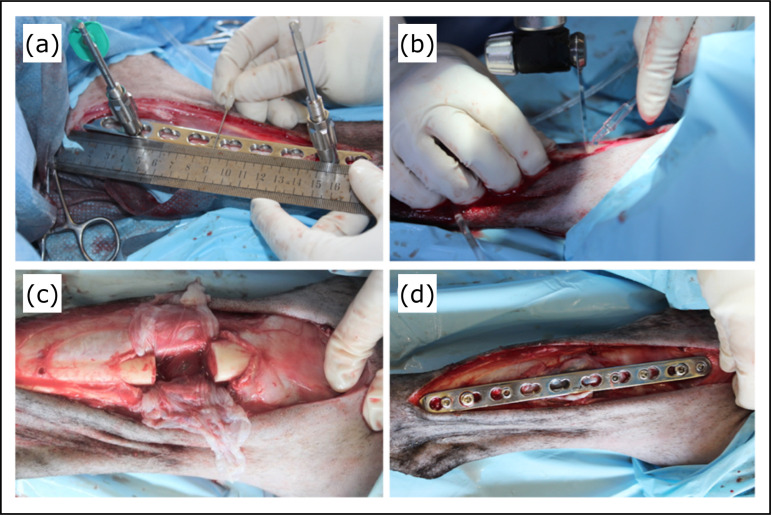
Intraoperative images of the ostectomy procedure. **(a)** Gap measurement prior to bone defect creation; **(b)** Bone transection using an oscillating saw; **(c)** Segmental bone defect; **(d)** Final appearance following completion of the osteosynthesis procedure.

Animals were submitted to a standardized surgical procedure consisting of creation of a 3-cm long bone defect in the right hind limb. Animals were placed in right lateral recumbency and the right leg clipped and scrubbed for aseptic surgery, first with 2% then with 0.5% chlorhexidine digluconate solution (Riohex 2% and Riohex 0.5%; Rioquímica, São José do Rio Preto, SP, Brazil). The limb was then draped and the tibia accessed via a dorsomedial approach. A 12-cm longitudinal skin incision was made and subcutaneous tissues gently dissected away. A 10-hole, 4.5-mm LCP (Focus, Indaiatuba, SP, Brazil) was contoured for anatomic fit on the medial aspect of the tibia using a bending template (DPS, Solna, Switzerland). The LCP construct is illustrated in Fig. 1. This plate was positioned 2.5 cm above the medial malleolus and anchored to the bone using two push-pull devices to determine the exact mid-shaft landmark (Fig. 2a). Proper plate position was confirmed with intraoperative radiographs (TR90 Min X Ray, Northbrook, EUA; Mark II G Sound Eklin, Carlsbad, CA, USA). The plate was then removed and parallel osteotomies performed 1.5 cm above and below the mid-shaft landmark to create a 3-cm long bone defect. This was done using a saline-cooled oscillating saw (DPS, Solna, Switzerland) in order to prevent overheating and bone necrosis (Fig. 2b). Surrounding soft tissues were also protected with wet sponges to avoid iatrogenic damage. The periosteum was stripped off transected bone ends to expose 1.5 cm of bare bone surface and fragments realigned and plated. The bone defect was then covered with a biological membrane (Fig. 2c).

The LCP was applied in bridging fashion, with four screws of appropriate length (Focus, Indaiatuba, SP, Brazil) inserted into the proximal and three into the distal (locked and cortical screw respectively) bone fragment (Fig. 2d). Subcutaneous tissues were closed with 2.0 polyglecaprone 25 (Monocryl Ethicon, São Paulo, SP, Brazil) using a modified double-layer Cushing pattern. The skin was apposed with 3.0 mononylon (Mononylon Ethilon Ethicon, São Paulo, SP, Brazil) in a simple continuous fashion. Finally, proper implant position and bone alignment were radiographically reconfirmed and a full limb cast applied (Hygia Cast, Woosam Medical Co, South Korea).

Postoperative pain management consisted of intravenous administration of 3 mg·kg^–1^ of tramadol hydrochloride (Tramadon, Cristália, Itapira, SP, Brazil) and 25 mg·kg^–1^ of dipyrone (D500, Zoetis, Campinas, SP, Brazil) three times a day for three days. Phenylbutazone (Equipalazone Injetável, Hertape Calier, Juatuba, SP, Brazil) and ranitidine hydrochloride (Teuto, Anápolis, GO, Brazil) were also given intravenously once a day for three days (4 and 2 mg·kg^–1^, respectively). Analgesic rescue consisting of intravenous administration of 0.2 mg·kg^–1^ of morphine hydrochloride (Dimorf, Cristália, Itapira, SP, Brazil) was used as needed. Antimicrobial therapy consisted of intramuscular administration of 2.2 mg·kg^–1^ of ceftiofur hydrochloride (Cef-50, Agener União, Embu-Guaçu, SP, Brazil) once a day for seven days. Serum alkaline phosphatase, calcium and phosphate levels were also measured.

Animals were confined in pairs throughout the experimental period. Physiological parameters (heart and respiratory rate, rectal temperature and ruminal motility), behavior indicators (reduced feed intake and rumination, reluctance to move or postural changes) and signs of lameness were assessed daily by the same large animal specialists in charge of preoperative care.

**Figure 3:**
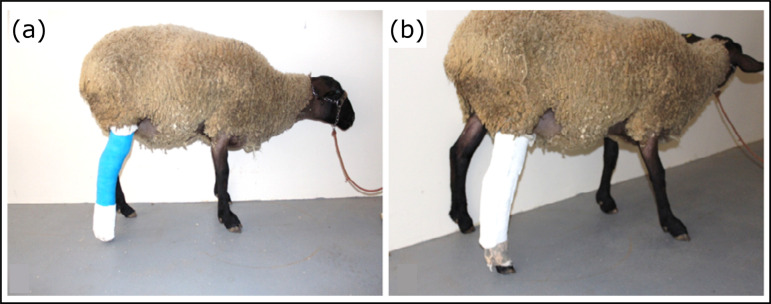
Postoperative immobilization. (a) Full limb cast; (b) Plantar splint allowing foot contact with the ground during weight bearing.

Full casts (Fig. 3a) were removed within 15 days of surgery for wound healing assessment and skin suture removal. Bivalve casts were then applied and changed on postoperative day 30. On postoperative day 60, bivalve casts were replaced by a plantar splint allowing foot contact with the ground during weight bearing (Fig. 3b). This was kept in place for the remaining 30 days of the experimental period. Radiographs were taken at 4-week intervals (postoperative days 30, 60 and 90).

Animals were euthanized on postoperative day 90 for histological and microtomographic analyses pertaining to other research projects. Euthanasia was achieved by injectable anesthetic overdose consisting of intravenous administration of 0.5 mg·kg^–1^ of 2% xylazine hydrochloride (Rompun, Bayer Saúde Animal, São Paulo, SP, Brazil) and 12.5 mg·kg^–1^ of sodium thiopental (Thiopentax, Cristália, Itapira, SP, Brazil), followed by intravenous infusion of 19.1% potassium chloride (Halex Istar, Goiânia, GO, Brazil) at sufficient doses to induce cardiorespiratory arrest.

## Results

Operative time ranged from 90 to 120 min. Surgical procedures and anesthetic recovery were uneventful. Following assisted recovery in a padded room, animals were taken back to their stalls. Animals tolerated casts well and showed no difficulties in rising from sternal recumbency. Surgical wound healing was uneventful. However, three animals had developed pressure sores at low pastern and hock level by postoperative day 30. These were treated with wound cleansing, topic application of antibiotic ointment and intramuscular administration of 0.1 mg·kg^–1^ of meloxicam (Maxicam 2%, Ouro Fino, Cravinhos, SP, Brazil) once a day for three days.

**Figure 4:**
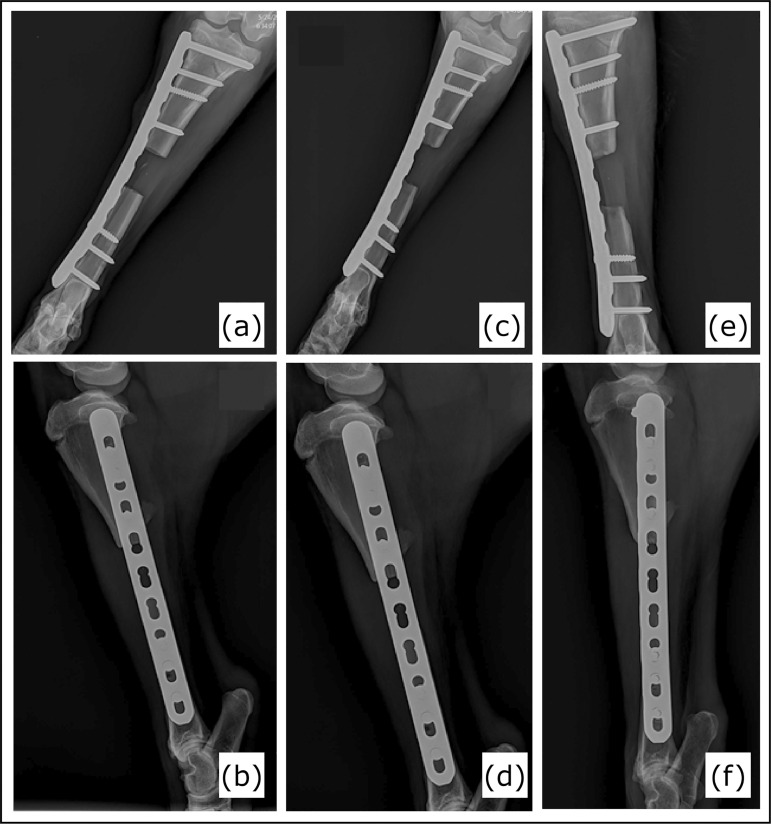
Postoperative radiographic images. Caudocranial (CC) and mediolateral (ML) views. Postoperative day 30: (a) CC view; (b) ML view. Postoperative day 60: (c) CC view; (d) ML view. Postoperative day 90: (e) CC view; (f) ML view.

Animals readily adapted to splints applied on postoperative day 60. Radiographic assessments and cast changes were carried out without sedation. Patients were manually restrained by caretakers and veterinarians in right lateral recumbency on a padded surface without expressing aggressive reactions. Follow-up radiographs revealed proper implant position and satisfactory bone healing with no implant failure, screw loosening or bone fracture/misalignment (Fig. 4). All animals recovered well from surgery, with no significant changes in physiological parameters or behavior and no signs of lameness over the course of the 90-day follow-up period.

## Discussion

The 3 cm segmental tibial bone defect model described in this study is widely known for providing appropriate critical-sized bone defects^19^. Bone defect length in this study exceeded 2 to 2.5 times the diameter of the operated bone and the periosteum was stripped off transected bone ends to prevent bone healing during the experimental period^1^,^20^. The 90-day follow-up period is also consonant with bone healing time in any given clinical fracture^21^.

Some guidelines must be followed for successful osteosynthesis in bone healing studies. The procedure must be technically feasible and repeatable. It must also replicate clinical conditions and be consistent with gait and biomechanical characteristics of the target species. Lastly, it should be amenable to imaging-based assessment while not compromising the well-being^21^–^23^ . In this study, the dorsomedial approach was selected to expose the middle third of the tibial shaft. The medial aspect of the tibia is thought to be a safe zone due to minimal soft tissue coverage and lack of important neurovascular structures. These anatomical features allow appropriate imaging assessment and greater exposure for surgical procedures, such as ostectomy, osteosynthesis and scaffold implantation or any other procedure involving bone defect treatment with biomaterial implants, with lower risk of iatrogenic damage 11,19.

Successful use of LCPs in animal models and clinical cases requires appropriate surgical planning. Gap size must be consistent with research goals. Implants must be made of appropriate material, both from a quality and a safety standpoint. Plate size, plate-to-bone distance and screw number, size and orientation must also be accounted for^24^. Most complications associated LCP fixation stem from technical errors such as implant size underestimation and inappropriate locking screw placement^16^. Preoperative radiographic assessment was vital for surgical planning in this study. Anatomical LCP contouring ensured long-lasting stable fixation^25^. Use of plates with combi-holes allowing insertion of cortical screws in this model enhanced angular stability, particularly near the diaphyseal bone defect, ensuring successful outcomes^14^,^16^.

Previous investigations in sheep have shown that LCP fixation of bone defects followed by full, unrestricted weight-bearing is associated with complications such as fractures and may compromise subject retention^1^. Studies reporting high success rates with this technique often involve the use of some sort of suspension device in the postoperative period^11^,^26^. These devices were not used in this study. Postoperative management consisted of limb casts for the first 60 days of the experimental period, followed by splints that did not prevent foot contact with the ground during weight bearing for the remaining 30 days. This strategy was thought to faithfully replicate real-life postoperative recovery in large animal orthopedics^18^.

Implant failure, infection and severe fractures requiring subject exclusion have been reported in bone tissue engineering studies involving external fixation or internal fixation with intramedullary pins in sheep^2^,^27^,^28^. Field and Ruthenbeck^23^ reported no complications following interlocking nail fixation in sheep. However, animals in their study were supported by a sling in the first three weeks after surgery. Dynamic compression plates have been widely used in sheep. Berner *et al.* 7 and Cipitria *et al.* 12 reported successful outcomes with no major complications following application of such plates. In those studies, operated limbs were not immobilized and animals were allowed to fully bear weight. However, complications such as infection, plate exposure and implant failure have been described^29^,^30^. Sparks *et al*.^19^ achieved high success rates with the use of DCP in sheep. However, authors of that study recommend plaster cast immobilization and sling suspension in the first four weeks of the postoperative period. Animals treated in this manner are able to deambulate, but not to lie down. Experimental design in this study was not disruptive to animal well-being. Confinement in well bedded stalls of sufficient size to accommodate two animals and postoperative weight bearing (i.e., no use of suspension devices) allowing unrestricted movement, rest and access to feed was thought to encourage normal animal behavior throughout the experimental period. Postoperative follow-up plays a major role in morbidity and mortality rates associated with this bone healing model. Animal studies should be designed in compliance with the 3R principle (replacement, reduction and refinement) to reduce and optimize animal use in scientific investigations^11^,^22^. Findings of this study suggest the method proposed promotes appropriate levels of well-being and can be applied to research in humans as well as veterinary orthopedics.

## Conclusions

Experimental creation of segmental tibial bone defects followed by locking compression plate fixation and postoperative cast and splint immobilization proved technically feasible in sheep. Combination of internal fixation and appropriate postoperative management increased construct stability and reduced the risk of postoperative complications. This strategy also allowed satisfactory postoperative recovery without sling suspension. The method proposed was thought to faithfully replicate real-life large animal orthopedics scenarios without compromising animal well-being.
